# Triple exponentially weighted moving average control chart with measurement error

**DOI:** 10.1038/s41598-023-41761-7

**Published:** 2023-09-07

**Authors:** Jing Wang, Muhammad Arslan, Afshan Riaz, Showkat Ahmad Lone, Nevine M. Gunaime

**Affiliations:** 1 Taiyuan Normal University, Jianzhong, China; 2 Jiangsu University, and Institute of Southern Punjab, Multan, Pakistan; 3 University of Sargodha, Sargodha Punjab, Pakistan; 4 Sudia Electronic University, Riyadh, Saudi Arabia

**Keywords:** Statistics, Mathematics and computing

## Abstract

Measurement error (M.E) can have a substantial impact on quality control applications, diminishing the sensitivity to detect changes in the mean or variance of quality characteristics. To monitor shifts in process mean and dispersion, Exponentially Weighted Moving Average (EWMA) and Cumulative Sum (CUSUM) control charts are commonly employed. In our research, we investigated the influence of M.E on the Triple Exponentially Weighted Moving Average (TEWMA) control chart. We assessed the performance of the control chart using Average Run Length (ARL) as the evaluation metric. To compute the ARL properties, we adopted the Monte-Carlo simulation method. A comparison section has been made to check the performance efficiency of the control chart with the existing EWMA control chart. The implementation of a control chart on a real data set is also presented.

## Introduction

The target of every manufacturing, industrial, and service sector is to produce quality products. Every producer desire to produce quality product by using minimum cost and effort. In statistical process control (SPC), the main objective is to detect the variation in the parameters of the production process. Under SPC, one of the prompt tools is control charts. A graphical presentation of the change in a process over time is called control charting. A control chart contains three lines *i.e.* lower, central, and upper lines. These lines are used to draw a conclusion about the process situation. By the characteristics, control charts are divided into two categories (i) attributed control charts and (ii) variable control charts. The variable control charts are further divided into (i) memory type and (ii) memory-less control charts. Shewhart control chart is an example of a memory-less control chart proposed by Shewhart in 1920. The EWMA and CUSUM control charts are an example of memory-type control charts. The EWMA control chart proposed by Ref.^1^ and Cusum control chart proposed by Ref.^2^. The memory-type control charts have more efficient performance than memory-less as they used current information along with previous information to conclude.

The measurements that are quantitative in nature are usually measured through any instruments. In real-life it is hardly possible to collect the hundred percent correct measurement, there is some extent from the actual value. Over the last few decades, the authors paid attention to this regard to study the effect of measurement on the performance of control charts. For instance, Refs.^3–9^. In the literature, different methods have been discussed such as (i) covariate model (ii) multiple measurements, and (iii) linearly increasing variance method. The authors reported that the performance of the control charts has been affected in the presence of M.E, as increasing in the run length properties.

The detection ability of memory-type control charts is more sensitive than memory-less control charts to detect a small change in the process parameters such as mean and variance. Various authors presented EWMA control charts with different statistics for improving the performance of control charts such as Ref.^10^ observed that the double EWMA (DEWMA) control chart has better performance than the EWMA control chart. Reference^11^ presented a DEWMA control chart. Reference^12^ presented DEWMA control chart with different run rules schemes for the process mean. Reference^13^ presented hybrid EWMA control chart in Bayesian approach. Reference^14^ discussed the control charting schemes by incorporating supplementary variables. Reference^15^ applied control chart in the field of health science. Later on, different authors worked on triple EWMA (TEWMA) control charts and reported that TEWMA control charts have better performance than traditional EWMA control charts. For instance, ^16^ proposed TEWMA control chart and reported that TEWMA control chart efficient than EWMA control chart. Similarly, Ref.^17^ presented TEWMA control chart for analyzed the time between events and compared it with DEWMA and traditional EWMA control charts. They concluded that the TEWMA control chart has better performance than DEWMA and EWMA control chart. Reference^18^ proposed TEWMA control chart for process dispersion. Recently, Ref,^19^ monitored the process location and sacle parameter by using distribution free TEWMA.

In this paper, we analyzed the effect of measurement of TEWMA control chart. Section “Measurement error”, based on the introduction of M.E. In Section “Proposed control chart”, we presented the proposed TEWMA control chart. Section “Performance evaluation”, presented the performance evaluation of the proposed control chart by using the Monte-Carlo simulation method. Section “Comparison”, presented the comparison between the proposed control chart and traditional EWMA control chart in the presence of M.E. The implementation of the proposed control chart on a real data set is presented in Section “Real-data application”. In the last, the conclusion has been discussed.

## Measurement error

Reference^20^ discussed the M.E variability that is frequently quantified by using the gage capability. The control chart is used to monitor the precision and accuracy of the measurement process over time using the observed deviation from the known standards. The concept of M.E in control charts was introduced by Ref.^21^ by using the model

1 $$ Y = X + \varepsilon , $$

where *X* is the value of the quality characteristic, and *Y* is the measured quantity. It is assumed that the *Y* and *X* are normally distributed with the same means but different variances. the variance of *X* is less than the variance of *Y,* because its variance comprises both the variances of *X* and $\varepsilon$. The random error due to measurement imprecision is denoted by $\varepsilon$ and it is assumed to be normally distributed with mean zero and variance $\sigma_{m}^{2}$. In the literature, three different methods have been discussed to evaluate the effect of M.E such as (i) the covariates (ii) multiple measurements, and (iii) linearly increasing variance methods.

### Covariates model

A covariate model introduced by Ref.^4^

2 $$ Y = A + BX + \varepsilon , $$

where $$Y\sim N\,\left( {A + B*\mu , \, B^{2} \sigma^{2} + \sigma_{m}^{2} } \right).$$

It is assumed that $A,B$,$\sigma^{2}$ and $\sigma_{m}^{2}$ are known, $A$ and $B$ are two constants. Model (1) is the special case of model (2) with $A = 0$ and $B = 1$.

### Multiple measurements

The multiple measurement methods in the M.E. introduced by Ref.^22^ to increase the statistical powers and decision by taking multiple measurements per sampling unit instead of taking a single measurement per item. The multiple measurements are used as a remedy to decrease the effect of M.E. The variance of the overall mean for multiple measurements with *K* (multiple measurements) is given by

3 $$ \frac{{B^{2} \sigma^{2} }}{n} + \frac{{\sigma_{m}^{2} }}{nk}. $$

By using the Model (2), the study variable $Y\sim N\,\left( {A + B*\mu , \, \frac{{B^{2} \sigma^{2} }}{n} + \frac{{\sigma_{m}^{2} }}{nk}} \right)$ in case of multiple measurements.

### Linearly increasing variance

It is observed that in many industrial problems, the variance of the process measurements depends on the mean level of the process. In this situation, the effect of M.E error can be studied by using the linearly increase variance method, where $Y\sim (A + B\mu , \, B^{2} \sigma^{2} + C + D\mu )$, where $C$ and $D$ are two known constants. The $D$ parameter has the additional effect of changing the process mean underlying measurements. it is assumed that $\varepsilon$ is distributed as normal with mean zero and variance $C + D\mu$^20^.

## Proposed control chart

Reference^16^ introduced TEWMA control chart by using the statistic

4 $$ \left\{ \begin{gathered} Z_{i} = \lambda Y_{i} + (1 - \lambda )Z_{i - 1} , \hfill \\ W_{i} = \lambda Z_{i} + (1 - \lambda )W_{i - 1} , \hfill \\ Y_{i} = \lambda W_{i} + (1 - \lambda )Y_{i - 1} , \hfill \\ \end{gathered} \right. $$

where $Z_{0} = W_{0} , \, Y_{0} = \mu_{0}$ and $\lambda$ is the smoothing parameter and it lies between zero and one. A rule of thumb is to use the small values of $\lambda$ to detect smaller shifts. The mean of the TEWMA statistic is defined as

$$ E(Y_{i} ) = \mu_{0} , $$

and variance ($Y_{i}$) is defined as

5 $$  \begin{aligned}   \sigma _{{Yi}}^{2}  & = \frac{{\theta ^{3} \lambda ^{6} }}{4}\left[ { - \left[ {\frac{{i(i^{2}  - 1)(i - 2)\theta ^{{i - 3}} }}{{1 - \theta }}} \right] - 4\left[ {\frac{{i\left( {i^{2}  - 1} \right)\theta ^{{i - 2}} }}{{(1 - \theta )^{2} }}} \right] - 12\left[ {\frac{{i(i + 1)\theta ^{{i - 1}} }}{{(1 - \theta )^{3} }}} \right] - 24\left[ {\frac{{(i + 1)\theta ^{i} }}{{(1 - \theta )^{4} }}} \right] + 24\left[ {\frac{{\left( {1 - \theta ^{{i + 1}} } \right)}}{{(1 - \theta )^{5} }}} \right]} \right] \\ & \quad +      2\theta ^{2} \lambda ^{6} \left[ { - \left[ {\frac{{i\left( {i^{2}  - 1} \right)\theta ^{{i - 2}} }}{{(1 - \theta )}}} \right] - 3\left[ {\frac{{i(i + 1)\theta ^{{i - 1}} }}{{(1 - \theta )^{2} }}} \right] - 6\left[ {\frac{{(i + 1)\theta ^{i} }}{{(1 - \theta )^{3} }}} \right] + 6\left[ {\frac{{\left( {1 - \theta ^{{i + 1}} } \right)}}{{(1 - \theta )^{4} }}} \right]} \right] \\ & \quad +  \frac{{7\theta \lambda ^{6} }}{2}\left[ { - \left[ {\frac{{i(i + 1)\theta ^{{i - 1}} }}{{(1 - \theta )}}} \right] - \left[ {\frac{{2(i + 1)\theta ^{i} }}{{(1 - \theta )^{2} }}} \right] + \left[ {\frac{{2\left( {1 - \theta ^{{t + 1}} } \right)}}{{(1 - \theta )^{3} }}} \right]} \right] + \lambda ^{6} \left[ {\left[ {\frac{{\left( {1 - \theta ^{{i + 1}} } \right)}}{{(1 - \theta )^{2} }} - \frac{{(i + 1)\theta ^{i} }}{{(1 - \theta )}}} \right]} \right], \hfill \\  \end{aligned}    $$

where, $$\theta = (1 - \lambda )^{2} .$$

By using the variance in (5) the time-varying control limits can be defined as

6 $$ UCL/LCL = \mu_{0} \pm L\sqrt {\sigma_{Yi}^{2} } ,\,\,\,\,\,CL = \mu_{0} . $$

For the large value of *i,* the variance of $Y_{i}$ can be defined as

7 $$ \sigma_{{Y_{i} }}^{2} = \frac{{6(1 - \lambda )^{6} \lambda }}{{(2 - \lambda )^{5} }} + \frac{{12(1 - \lambda )^{4} \lambda^{2} }}{{(2 - \lambda )^{4} }} + \frac{{7(1 - \lambda )^{2} \lambda^{3} }}{{(2 - \lambda )^{3} }} + \frac{{\lambda^{4} }}{{(2 - \lambda )^{2} }}. $$

The control limits can be written asymptotically by using the variance in (7).

8 $$ UCL/LCL = \mu_{0} \pm L\sigma \sqrt {\frac{{6(1 - \lambda )^{6} \lambda }}{{(2 - \lambda )^{5} }} + \frac{{12(1 - \lambda )^{4} \lambda^{2} }}{{(2 - \lambda )^{4} }} + \frac{{7(1 - \lambda )^{2} \lambda^{3} }}{{(2 - \lambda )^{3} }} + \frac{{\lambda^{4} }}{{(2 - \lambda )^{2} }}} , $$

For more detail see Ref.^16^.

In this study, we have presented TEWMA control chart with M.E. The TEWMA statistic with covariates model is defined as

9 $$ \left\{ \begin{gathered} Z_{i} = \lambda Y_{i} + (1 - \lambda )Z_{i - 1} , \hfill \\ W_{i} = \lambda Z_{i} + (1 - \lambda )W_{i - 1} , \hfill \\ X_{i} = \lambda W_{i} + (1 - \lambda )X_{i - 1} , \hfill \\ \end{gathered} \right. $$

where $Z_{0} = W_{0} , \, Y_{0} = A + B\mu_{0}$ and the control limits can be written as

10 $$ UCL/LCL = \mu_{0} \pm L\sqrt {B^{{^{2} }} \left( {\frac{{6(1 - \lambda )^{6} \lambda }}{{(2 - \lambda )^{5} }} + \frac{{12(1 - \lambda )^{4} \lambda^{2} }}{{(2 - \lambda )^{4} }} + \frac{{7(1 - \lambda )^{2} \lambda^{3} }}{{(2 - \lambda )^{3} }} + \frac{{\lambda^{4} }}{{(2 - \lambda )^{2} }}} \right) + \sigma_{m}^{2} } . $$

The control limits for multiple measurements and linearly increasing variance methods are respectively given as

11 $$ UCL/LCL = \mu_{0} \pm L\sqrt {\frac{{B^{2} }}{n}\left( {\frac{{6(1 - \lambda )^{6} \lambda }}{{(2 - \lambda )^{5} }} + \frac{{12(1 - \lambda )^{4} \lambda^{2} }}{{(2 - \lambda )^{4} }} + \frac{{7(1 - \lambda )^{2} \lambda^{3} }}{{(2 - \lambda )^{3} }} + \frac{{\lambda^{4} }}{{(2 - \lambda )^{2} }}} \right) + \frac{{\sigma_{m}^{2} }}{nk}} . $$

12 $$ UCL/LCL = \mu_{0} \pm L\sqrt {B^{{^{2} }} \left( {\frac{{6(1 - \lambda )^{6} \lambda }}{{(2 - \lambda )^{5} }} + \frac{{12(1 - \lambda )^{4} \lambda^{2} }}{{(2 - \lambda )^{4} }} + \frac{{7(1 - \lambda )^{2} \lambda^{3} }}{{(2 - \lambda )^{3} }} + \frac{{\lambda^{4} }}{{(2 - \lambda )^{2} }}} \right) + C + D\mu } . $$

## Performance evaluation

There are different performance-measuring tools to evaluate the performance of control charts, *ARLs,* and *SDRLs* are famous among them. In the literature, Monte-Carlo simulation methods, Markov chain, and integral equation methods are available for computing the *ARLs* and *SDRLs*. With the minimum run lengths, the control chart is considered to be efficient or superior. For computing the run length properties we used the Monte-Carlo simulation method with 1,000000 iterations. This simulation method to evaluate the out-of-control *ARLs* is described as follows:

1. A random sample of size 5 is generated with normal distribution with $\mu_{0} = 0, \, \sigma^{2} = 1$ such that $Y\sim N\left( {A + B\mu_{0} , \, B^{2} \sigma^{2} + \sigma_{m}^{2} } \right)$.
2. Calculate $Z_{i}$ for each subgroup.
3. For each subgroup calculate $W_{i}$, where $Z_{i}$ is the statistic of EWMA control chart.
4. Compute the value of $X_{i}$, where $X_{i}$ is the TEWMA Statistic
5. Compute the control limits defined in (10), (11), and (12) for respective M.E methods
6. Compared the statistics of the proposed control chart with respective control limits.
7. If the process is declared as in-control, again start from step 1, and repeat this procedure until the process is declared as out-of-control.
8. When the process is declared as out-of-control, record the in-control number of subgroups as run length.

The results are presented in Tables 1, 2, 3, 4, 5 and 6. The results of the proposed TEWMA control chart with and without considering the effect of M.E are presented in Table 1 for various values of shifts in mean such as 0.0, 0.05, 0.1, 0.25, 0.5, 0.75, 1, 1.5, 2, and 3 with $\lambda = 0.05$.

**Table 1:** *ARLs(SDRLs)* of TEWMA Control Chart with Covariate Model with different values of error ratio.

Δ	Sigm = 0.0	Sigm = 0.1	Sigm = 0.2	Sigm = 0.5	Sigm = 1
0.0	499.71 (458.78)	506.83 (474.16)	502.29 (462.28)	497.66 (460.73)	502.11 (456.18)
0.05	213.21 (173.96)	224.95 (182.83)	235.23 (195.30)	263.38 (221.19)	294.53 (257.11)
0.1	93.78 (56.62)	99.05 (61.51)	105.33 (68.30)	121.16 (83.35)	143.09 (103.52)
0.25	39.37 (8.78)	40.58 (9.45)	42.21 (10.61)	45.53 (13.17)	51.59 (17.76)
0.5	26.29 (2.84)	26.90 (3.03)	27.63 (3.23)	29.30 (3.81)	31.72 (4.83)
0.75	21.53 (1.61)	22.04 (1.72)	22.52 (1.82)	23.72 (2.12)	25.57 (2.60)
1	18.85 (1.11)	19.26 (1.19)	19.69 (1.26)	20.69 (1.42)	22.20 (1.74)
1.5	15.77 (0.70	16.09 (0.72)	16.42 (0.77)	17.23 (2.85)	18.39 (1.01)
2	13.96 (0.51)	14.21 (0.52)	14.51 (0.57)	15.20 (0.63)	16.20 (0.74)
3	11.88 (0.34)	12.02 (0.27)	12.19 (0.40)	12.86 (2.41)	13.63 (0.52)

**Table 2:** *ARLs(SDRLs)* of TEWMA Control Chart with Covariate Model with different values of *B.*

Δ	Sigm = 0.0	B = 1	B = 2	B = 3	B = 5
0.0	499.71 (458.78)	502.11 (456.18)	502.81 (471.21)	504.52 (464.91)	499.74 (463.77)
0.05	213.21 (173.96)	294.53 (257.11)	240.12 (199.47)	223.75 (188.41)	217.48 (177.39)
0.1	93.78 (56.62)	143.09 (103.52)	107.77 (70.31)	100.84 (62.85)	96.50 (59.44)
0.25	39.37 (8.78)	51.59 (17.76)	42.53 (10.83)	40.83 (9.32)	39.89 (8.93)
0.5	26.29 (2.84)	31.72 (4.83)	27.88 (3.32)	27.04 (3.08)	26.53 (2.90)
0.75	21.53 (1.61)	25.57 (2.60)	22.66 (1.84)	22.10 (1.72)	21.70 (1.65)
1	18.85 (1.11)	22.20 (1.74)	19.80 (1.27)	19.35 (1.18)	18.99 (1.13)
1.5	15.77 (0.70	18.39 (1.01)	16.55 (0.78)	16.14 (0.74)	15.89 (0.71)
2	13.96 (0.51)	16.20 (0.74)	14.62 (0.59)	14.27 (0.54)	14.05 (0.51)
3	11.88 (0.34)	13.63 (0.52)	12.29 (0.45)	12.04 (0.28)	11.93 (0.28)

**Table 3:** *ARLs(SDRLs)* of TEWMA Control Chart with Multiple Measurements at different values of error ratio.

Δ	Sigm = 0.0	Sigm = 0.1	Sigm = 0.2	Sigm = 0.5	Sigm = 1
0.0	499.71 (458.78)	500.82 (468.52)	505.63 (470.28)	503.50(465.51)	495.83(456.34)
0.05	213.21 (173.96)	83.05 (46.15)	83.56 (46.77)	87.33 (50.75)	91.00 (53.77)
0.1	93.78 (56.62)	43.059 (11.04)	43.11 (11.03)	44.23 (11.95)	45.78 (13.05)
0.25	39.37 (8.78)	24.99 (2.42)	25.15 (2.50)	25.46 (2.58)	26.02 (2.71)
0.5	26.29 (2.84)	18.02 (0.98)	18.09 (0.98)	18.32(1.04)	18.69 (1.07)
0.75	21.53 (1.61)	15.09 (0.62)	15.15 (0.62)	15.35 (0.64)	15.62 (0.67)
1	18.85 (1.11)	13.364 (0.50)	13.41 (0.51)	13.59 (0.52)	13.83 (0.51)
1.5	15.77 (0.70	11.21 (0.40)	11.27 (0.44)	11.49 (0.50)	11.77 (0.42)
2	13.96 (0.51)	10.00 (0.07)	10.01 (0.10)	10.06 (0.24)	10.30 (0.46)
3	11.88 (0.34)	8.77 (0.41)	8.87 (0.32)	8.98 (0.12)	8.99 (0.01)

**Table 4:** *ARLs(SDRLs)* of TEWMA Control Chart with Multiple Measurements at different values of *K*.

Δ	Sigm = 0.0	K = 5	K = 10	K = 20	K = 50
0.0	499.71 (458.78)	495.83 (456.34)	500.24 (466.64)	501.38 (457.61)	501.80 (463.64)
0.05	213.21 (173.96)	91.00 (53.77)	87.45 (50.36)	86.32 (50.43)	83.51 (47.10)
0.1	93.78 (56.62)	45.78 (13.05)	44.32 (11.84)	43.43 (11.16)	42.82 (10.88)
0.25	39.37 (8.78)	26.02 (2.71)	25.50 (2.60)	25.13 (2.50)	25.01 (2.46)
0.5	26.29 (2.84)	18.69 (1.07)	18.34 (1.03)	18.14 (1.01)	18.02 (0.98)
0.75	21.53 (1.61)	15.62 (0.67)	15.35 (0.64)	15.21 (0.62)	15.10 (0.62)
1	18.85 (1.11)	13.83 (0.51)	13.60 (0.52)	13.44 (0.51)	13.35 (0.50)
1.5	15.77 (0.70	11.77 (0.42)	11.48 (0.500)	11.30 (0.45)	11.21 (0.41)
2	13.96 (0.51)	10.30 (0.46)	10.06 (0.24)	10.01 (0.133)	10.00 (0.08)
3	11.88 (0.34)	8.99 (0.01)	8.98 (0.12)	8.90 (0.29)	8.79 (0.40)

**Table 5:** *ARLs(SDRLs)* of TEWMA Control Chart with Linearly increasing variance at different values of *D*.

Δ	Sigm = 0.0	D = 1	D = 2	D = 3	D = 5
0.0	499.71 (458.78)	505.27 (464.10)	499.15 (459.54)	505.22 (459.80)	500.42 (464.90)
0.05	213.21 (173.96)	454.64 (412.58)	468.46 (429.89)	482.66 (441.59)	484.33(437.74)
0.1	93.78 (56.62)	333.43 (296.12)	398.10 (357.93)	439.18(401.38)	452.82(415.53)
0.25	39.37 (8.78)	133.74 (95.23)	195.64 (157.91)	237.67 (199.85)	297.42(215.85)
0.5	26.29 (2.84)	59.97 (25.20)	84.52 (47.00)	107.18 (68.44)	144.54(107.13)
0.75	21.53 (1.61)	42.15 (10.49)	55.57 (21.08)	67.49 (31.53)	88.56 (51.22)
1	18.85 (1.11)	34.89 (6.34)	43.47 (11.44)	51.00 (17.33)	64.39 (28.75)
1.5	15.77 (0.70	27.72 (3.26)	33.32 (5.59)	37.64 (7.75)	44.53 (12.06)
2	13.96 (0.51)	23.94 (2.17)	28.22 (3.44)	31.50 (4.61)	36.50 (7.03)
3	11.88 (0.34)	19.75 (1.25)	22.96 (1.94)	25.35 (2.53)	28.77 (3.68)

**Table 6:** *ARLs(SDRLs)* of TEWMA Control Chart with Linearly increasing variance at different values of *C*.

Δ	Sigm = 0.0	C = 0	C = 1	C = 2	C = 3
0.0	499.71 (458.78)	505.27 (464.10)	500.19 (465.09)	501.91 (461.83)	506.36 (468.83)
0.05	213.21 (173.96)	454.64 (412.58)	451.14 (405.88)	439.63 (400.86)	454.47 (409.12)
0.1	93.78 (56.62)	333.43 (296.12)	341.77 (300.13)	351.71 (311.02)	350.06 (311.38)
0.25	39.37 (8.78)	133.74 (95.23)	139.36 (100.29)	146.24 (107.59)	154.72 (115.30)
0.5	26.29 (2.84)	59.97 (25.20)	62.68 (27.20)	65.28 (29.78)	67.91 (31.63)
0.75	21.53 (1.61)	42.15 (10.49)	43.63 (11.74)	45.15 (12.93)	46.46 (13.97)
1	18.85 (1.11)	34.89 (6.34)	35.90 (6.70)	36.85 (7.26)	37.73 (7.80)
1.5	15.77 (0.70	27.72 (3.26)	28.40 (3.50)	29.02 (3.73)	29.62 (3.93)
2	13.96 (0.51)	23.94 (2.17)	24.48 (2.31)	24.98 (2.47)	25.42 (2.52)
3	11.88 (0.34)	19.75 (1.25)	20.14 (1.32)	20.50 (1.40)	20.90 (1.47)

The results related to the covariate method are presented in Tables 1, 2. In Table 1, the results are given for different values of ${{\sigma_{m}^{2} } \mathord{\left/ {\vphantom {{\sigma_{m}^{2} } {\sigma_{{}}^{2} }}} \right. \kern-0pt} {\sigma_{{}}^{2} }}$ by taking *A* = 0 and $B = 1$. The results in Table 2 are obtained by using the same parameters but different values of *B* keeping ${{\sigma_{m}^{2} } \mathord{\left/ {\vphantom {{\sigma_{m}^{2} } {\sigma_{{}}^{2} }}} \right. \kern-0pt} {\sigma_{{}}^{2} }} = 1.$ The following observations are acquired from the results using the covariate method presented in Tables 1, 2.

(i) As expected, the *ARL* and *SDRL* values are increasing by an increase in the ratio ${{\sigma_{m}^{2} } \mathord{\left/ {\vphantom {{\sigma_{m}^{2} } {\sigma_{{}}^{2} }}} \right. \kern-0pt} {\sigma_{{}}^{2} }}$. This indicates the adverse effect of measurement error on the performance of TEWMA control chart. The results in Table 1 are similar to Ref.^5^*.*
(ii) It is observed from Table 2 that values of *ARL* and *SDRL* decrease as the *B* increases so the effect of measurement error is decreasing by increasing the value of *B*. It can be concluded that the effect of M.E is reduced by values of *B.*

The results related to the multiple measurements method are presented in Tables 3, 4. In Table 3, the results are given for different values of ${{\sigma_{m}^{2} } \mathord{\left/ {\vphantom {{\sigma_{m}^{2} } {\sigma_{{}}^{2} }}} \right. \kern-0pt} {\sigma_{{}}^{2} }}$ with *A* = 0, $B = 1$ and *k* = 5. In Table 4, the results are obtained using ${{\sigma_{m}^{2} } \mathord{\left/ {\vphantom {{\sigma_{m}^{2} } {\sigma_{{}}^{2} }}} \right. \kern-0pt} {\sigma_{{}}^{2} }} = 1$ and *B* = 1 but different values of *k.* The following observations are acquired from the results using the multiple measurements method.

(i) As expected, the *ARL* and *SDRL* values are increasing by increasing the error ratio.
(ii) The effect of measurement error is decreased by increasing the value of *B*.
(iii) From Table 5, the values of *ARL* and *SDRL* decrease as the *k* increases. The effect of measurement error is decreased by increasing the value of *k.* The results in (i), (ii) and (iii) are according to Ref.^5^*.*

In Table 5, the results are given for different values of $D$ with *A* = 0, $B = 1$, *C* = 0 and ${{\sigma_{m}^{2} } \mathord{\left/ {\vphantom {{\sigma_{m}^{2} } {\sigma_{{}}^{2} }}} \right. \kern-0pt} {\sigma_{{}}^{2} }} = 1$. The results in Table 6 are obtained using the same parameters but different values of *C* keeping ${{\sigma_{m}^{2} } \mathord{\left/ {\vphantom {{\sigma_{m}^{2} } {\sigma_{{}}^{2} }}} \right. \kern-0pt} {\sigma_{{}}^{2} }} = 1$, $B = 1$ and $D = 1$. The following observations are acquired from the results using the linearly increasing variance method presented in Tables 5, 6.

i) In Table 5, *ARL* and *SDRL* values are increasing by an increase in the values of *D,* so an increasing effect is observed by an increase in *D*.
ii) It is observed from Table 6, that values of *ARL* and *SDRL* increase as increases in *C*. The effect of M.E is increasing by an increase in the value of *C*.

## Comparison

In this section, we compare the performance of the proposed control chart with the traditional EWMA control chart in the presence of M.E. For the valid comparison, we fixed the in-control *ARL* = 500 at λ = 0.05, with *n* = 5. Table 7 provided the *ARLs* and *SDRLs* of proposed control chart and existing EWMA control chart by using covariates method and multiple measurements method. From the results, it is observed that at small shifts the *ARLs* and *SDRLs* are decreases with the proposed control chart and for large shifts the EWMA has minimum *ARLs*. From the results, it is concluded that the proposed control chart has better performance than existing EWMA control chart for small shifts in the presence of M.E.

**Table 7:** Comparison through *ARLs (SDRLs)* of EWMA and TEWMA Control Charts with M.E.

δ	Covariates model		Multiple measurements	
	EWMA	TEWMA	EWMA	TEWMA
0.0	495.40 (482.10)	502.11 (456.18)	506.38 (485.90)	497.56 (457.93)
0.025	436.69 (422.16)	425.43 (387.25)	244.29 (225.30)	208.09 (171.88)
0.05	332.72 (315.30)	294.53 (257.11)	96.40 (79.06)	91.81 (55.59)
0.075	233.27 (217.81)	203.24 (163.52)	51.49 (36.02)	58.46 (23.53)
0.1	164.72 (148.35)	143.09 (103.52)	33.06 (20.23)	45.71 (13.23)
0.25	41.42 (27.53)	51.59 (17.76)	9.74 (3.30)	26.02 (2.74)
0.5	15.26 (6.60)	31.72 (4.83)	4.57 (1.03)	18.68 (1.08)

## Real-data application

**Figure 1:**
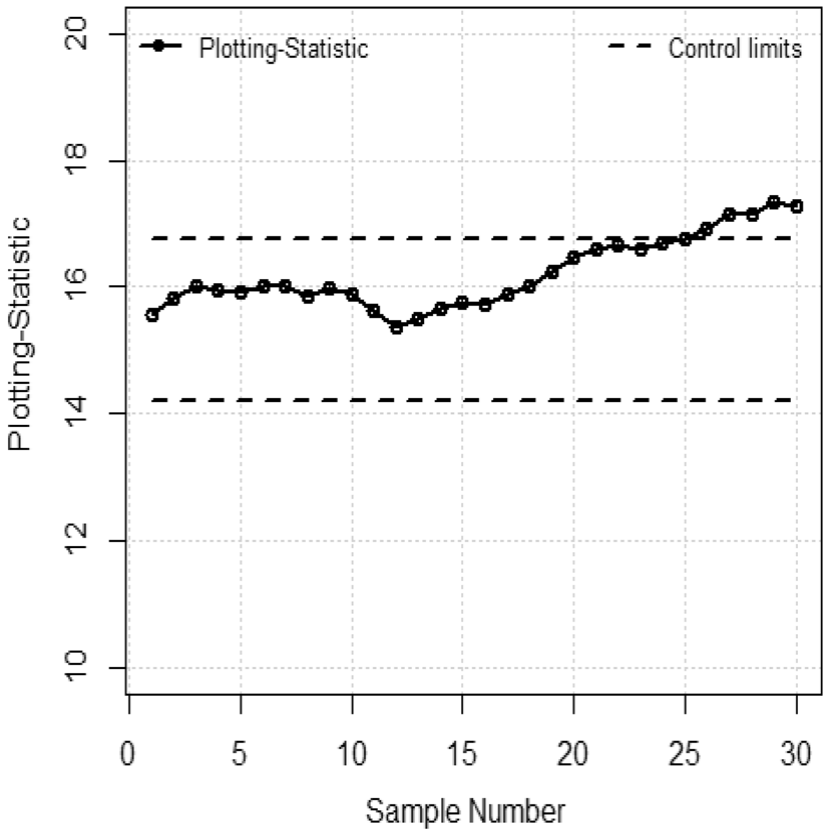
EWMA control chart with M.E.

**Figure 2:**
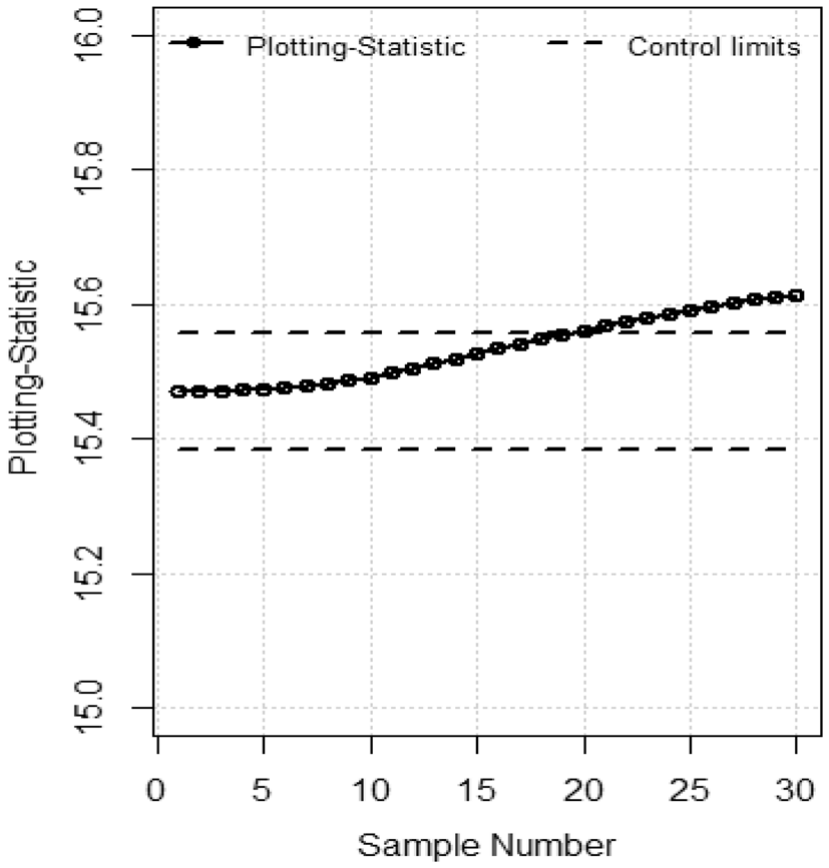
TEWMA control chart with M.E.

In this section an example is provided with available dataset of density where density is the study variable^23^. The population consists of 30 values with parameters $\mu_{y}$ = 15.47, $\sigma_{y}^{2}$ = 34.023, The 20 samples are taken from an in-control process and last 10 samples are taken when process is out-of-control with shift $0.05 \times \sigma_{y}^{{}}$. For the comparison purpose we fixed $ARL = 370$, $\lambda = 0.05$ and $\text{ratio = }1$ for both control charts. Figure 1 presents EWMA control chart with and $L = 2.325$, where Fig. 2 presents TEWMA control chart $L = 0.149$. In Fig. 1, the EWMA control chart with measurement error detected the first out-of-control point at 25th observations. In Fig. 2, the TEWMA control chart with measurement error detected the first out-of-control point at 20th observation so it is concluded that EWMA-Z chart has better performance than EWMA in the presence of measurement error (Table 8).

**Table 8:** The respective Statistics, lower and Upper control limits of EWMA, TEWMA Control Charts for data set.

S#	EWMA			TEWMA		
	Statistic	LCL	UCL	Statistic	LCL	UCL
1	15.56	14.19	16.74	15.47	15.38	15.55
2	15.82	14.19	16.74	15.47	15.38	15.55
3	16.02	14.19	16.74	15.47	15.38	15.55
4	15.96	14.19	16.74	15.47	15.38	15.55
5	15.93	14.19	16.74	15.47	15.38	15.55
6	16.02	14.19	16.74	15.47	15.38	15.55
7	15.99	14.19	16.74	15.47	15.38	15.55
8	15.84	14.19	16.74	15.48	15.38	15.55
9	15.97	14.19	16.74	15.48	15.38	15.55
10	15.88	14.19	16.74	15.49	15.38	15.55
11	15.62	14.19	16.74	15.49	15.38	15.55
12	15.36	14.19	16.74	15.50	15.38	15.55
13	15.49	14.19	16.74	15.51	15.38	15.55
14	15.65	14.19	16.74	15.51	15.38	15.55
15	15.76	14.19	16.74	15.52	15.38	15.55
16	15.71	14.19	16.74	15.53	15.38	15.55
17	15.87	14.19	16.74	15.54	15.38	15.55
18	16.02	14.19	16.74	15.54	15.38	15.55
19	16.24	14.19	16.74	15.55	15.38	15.55
20	16.45	14.19	16.74	**15.56**	15.38	15.55
21	16.59	14.19	16.74	**15.56**	15.38	15.55
22	16.67	14.19	16.74	**15.57**	15.38	15.55
23	16.58	14.19	16.74	**15.57**	15.38	15.55
24	16.69	14.19	16.74	**15.58**	15.38	15.55
25	**16.75**	14.19	16.74	**15.59**	15.38	15.55
26	**16.93**	14.19	16.74	**15.59**	15.38	15.55
27	**17.13**	14.19	16.74	**15.60**	15.38	15.55
28	**17.14**	14.19	16.74	**15.60**	15.38	15.55
29	**17.35**	14.19	16.74	**15.60**	15.38	15.55
30	**17.26**	14.19	16.74	**15.61**	15.38	15.55

Bold values indicate out-of-control observations.

## Conclusion

In this paper, we have studied the effect of M.E on the TEWMA control chart by assuming the covariate model. It is found that the M.E can affect the performance of the control charts by increasing the run length properties. Multiple measurements reduced the effect of M.E so it is used as a remedy of M.E. However, for multiple measurements, we need extra time and cost which is also a problem and for the solution to this problem, we need to design an economic study properly.

The linearly increasing variance another type of M.E also discussed and proved that affects the performance of the chart to a larger extent. We concluded that from Sections “Comparison” and “Real-data application”, the proposed control chart has efficient performance in the presence of M.E in the case of *ARLs* and as applied in real-life situations where the measurements are taken into account as ratio and interval scales.

## Data Availability

The datasets used and/or analysed during the current study available from the corresponding author on reasonable request.

## Abbreviations

M.E: Measurement error
SPC: Statistical process control
ARL: Average run length
SDRL: Standard deviation of run length
EWMA: Exponentially weighted moving average
CUSUM: Cumulative sum
TEWMA: Triple exponentially weighted moving average
DEWMA: Double exponentially weighted moving average
